# Future changes in the intensity and frequency of precipitation extremes over China in a warmer world: Insight from a large ensemble

**DOI:** 10.1371/journal.pone.0252133

**Published:** 2021-05-24

**Authors:** Yang Li, Jingyi Bai, Zhiwei You, Jun Hou, Wei Li

**Affiliations:** 1 Jiangsu Meteorological Observatory, Nanjing, China; 2 Jiangsu Meteorological Service Center, Nanjing, China; 3 Beijing PRESKY Technology Co., Ltd., Beijing, China; 4 Joint International Research Laboratory of Climate and Environment Change, Collaborative Innovation Center on Forecast and Evaluation of Meteorological Disaster, Nanjing University of Information Science and Technology, Nanjing, China; Texas A&M University, UNITED STATES

## Abstract

Sufficient samples of extreme precipitation events are needed in order to obtain reliable estimates of the probability of their occurrence. Here, we use a large ensemble simulation with 50 members from the Canadian Earth System Model (CanESM2) under the representative concentration pathway 8.5 (RCP8.5) scenario to give future projection of the intensity and frequency of extreme precipitation events under different warming levels relative to the current climate over China. A bias-correction method based on quantile mapping is first used to remove systematic biases in the ensemble. The return value and return period are obtained by fitting enough annual maximum precipitation samples with the generalized extreme value to represent the intensity and frequency of extreme events, respectively. The results show that the average intensity of extreme precipitation in China will increase by nearly 8% per 1°C of global warming, which closely follows the Clausius–Clapeyron relation. Rarer extreme events will experience greater changes in frequency, especially under higher warming. The nationally averaged extreme precipitation events, presently expected to occur every 50 years (100 years) under the current climate conditions, are expected to occur approximately every 41 years (82 years), 32 years (62 years), 22 years (42 years) and 15 years (29 years) under warming levels of 1.5, 2.0, 3.0 and 4.0°C, respectively. Northwestern China (NW), southwestern China (SW) and the Yangtze River valley (YZ) exhibit the greatest increase in probability ratio (PR) under future climate condition. The risk of extreme precipitation events, currently expected to occur once every 50 years, will be nearly 11 (21) times more likely to occur under a climate warming by 3.0°C (4.0°C). Limiting warming to 1.5°C will help avoid approximately 40%-50%, 70%-80% and over 90% of the increase in the risk of extreme events in almost all subregions if the global mean surface temperature (GMST) continues warming to 2.0°C, 3.0°C and 4.0°C, respectively. Our study provides a useful information for the understanding the impact of climate change on the future risk of extreme events over China.

## Introduction

In recent decades, numerous record-breaking extreme precipitation events have occurred around the world, including in many regions of China. These events pose serious threats to life and property. For example, in 2018 a record-breaking persistent heavy rainfall event that occurred over central western China affected 2.9 million people and resulted in a reported direct economic loss of over 8.9 billion yuan (National Disaster Reduction Commission; https://reliefweb.int/disaster/tc-2018-000110-chn). In May 2016, the lower reaches of the Yangtze River valley experienced extreme rainfall that broke the 56-year daily maximum records at 25 stations. These extreme events lead to waterlogging, landslides, and other disasters, resulting in severe damage to crops and disruption of agricultural production [1]. Thus, the future projection of such extreme precipitation events is of great concern to policy-makers and the general public.

It has been reported that the intensity and frequency of extreme events will continue to increase as the GMST continues to increase. Increasing air temperature moistens the atmosphere and therefore alters the hydrological cycle. This higher level of atmospheric moisture can produce more intense precipitation. It has been reported that the intensification of extreme precipitation events roughly follows the Clausius Clapeyron rate of an increase in moisture by 6%~7% per 1°C increase in temperature (Dai et al., 2006; Zhao et al., 2012), which is approximately the same rate as that of the moistening of the atmosphere due to warming [2]. This scaling value is found in observed in observations and simulations in global climate models (GCMs).

GCMs are the main tools used for projecting future changes in extreme precipitation events [3,4]. Many studies have used Phase 5 models of the Coupled Model Intercomparison Project (CMIP5) from the World Climate Research Program (WCRP) to investigate future changes in extreme precipitation because these models have performed relatively well in simulating observed extreme precipitation over China [5,6]. For example, the intensity of extreme precipitation increases by approximately 7% and 11%, respectively, at global warming of 1.5°C and 2°C. The probability of a 100-year historical event is projected to increase by a factor of 1.6 and 2.4, respectively, at the 1.5°C and 2°C global warming levels, respectively [5]. However, previous works have been based mainly on the multi-model ensemble from CMIP5. Diversities among models can be quite large, which may be inappropriate when combining extreme samples from individual models together.

Here we extract the extreme samples from a large ensemble simulation using a single GCM. The use of a large climate model ensemble allows us to directly estimate the probability of rare events (e.g., those with return intervals as high as 100-years). Large “initial-condition” ensemble experiments have been developed in recent years and are widely used to research extreme events [7–10]. Ensemble members from each experiment are subjected to the same external forcing but are started from different initial conditions. Multiple realizations from one model can provide sufficient sampling of extreme precipitation events to study future changes in such events. Therefore, this paper focus on the changes in extreme precipitation events in terms of intensity and frequency under different global warming levels over China using a large ensemble simulation, which may be an important supplement to research on the future risk of extreme precipitation events over China.

The structure of this paper is as follows: section 2 and section 3 introduce the data and methods, respectively. The projected changes in extreme precipitation are presented in section 4, followed by a discussion in section 5. Finally, section 6 summarizes the conclusions.

## Data

### Observation data

**Fig 1:**
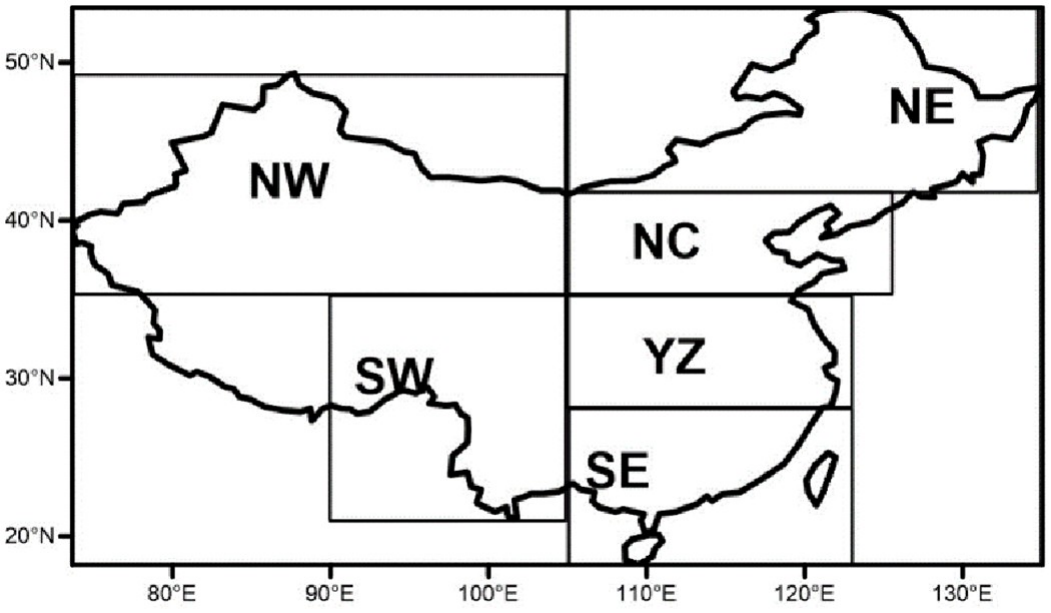
Map showing the 543 stations in this study and the six subregions of China: Northwestern China (NW: 35°–50°N, 74°–105°E), southwestern China (SW: 20°–35°N, 90°–105°E), northeastern China (NE: 42°–50°N, 105°–134°E), northern China (NC: 35°–42°N, 105°–125°E), the Yangtze River valley (YZ: 28°–35°N, 105°–123°E), and southeastern China (SE: 18°–28°N, 105°–120°E). (Made with Natural Earth. Free vector and raster map data @ naturalearthdata.com).

Daily precipitation data from 1961 to 2017 collected by the CMA (http://cdc.nmic.cn/home.do) at 726 meteorological stations were used in this research. Rigorous quality control procedures have been applied to this dataset by the National Meteorological Information Center [11]. Of the total, 543 stations have been used because these showed no missing values in the record for any year during this period (Fig 1).

Climate characteristics over China vary significantly from region to region. To better understand the potential future changes in extreme precipitation in different regions, we divided the whole of China into six subregions (Fig 1): northwestern China (NW: 35°–50°N, 74°–105°E), southwestern China (SW: 20°–35°N, 90°–105°E), northeastern China (NE: 42°–50°N, 105°–134°E), northern China (NC: 35°–42°N, 105°–125°E), the Yangtze River valley (YZ: 28°–35°N, 105°–123°E), and southeastern China (SE: 18°–28°N, 105°–120°E). The distribution of subregions has been widely used in previous studies to demonstrate the regional features of climate change [12,13].

### Model dataset

We used a large initial-condition ensemble to robustly assess future change in intensity and frequency of extreme precipitation events over China under different warming levels. Ensemble members from each experiment are subjected to the same external forcing but are started from different initial conditions. This ensemble was conducted with the Canadian Earth System Model (CanESM2) at spatial resolution of 2.8° × 2.8°, referred to hereafter as the CanESM2 large ensemble (CanESM2-LE). CanESM2 combines atmosphere, ocean, land-surface, sea ice, and carbon-cycle components in a coupled framework in which all components interact. The atmosphere component (CanAM4) has evolved from the third-generation atmospheric general circulation model (CanAM3). Key new features in CanAM4 include a new radiative transfer scheme [14], a prognostic bulk aerosol scheme considering the direct and indirect radiative of aerosol [15], a fully cloud microphysics scheme [16] and a new shallow convention scheme [17]. A more detailed description of CanESM2 is given in Arora et al. (2011). CanESM2-LE consists of 50 members for the 1950 to 2100 period, each of these was subject to identical external radiative forcing but beginning from slightly different atmospheric initial conditions [18,19]. Historical natural and anthropogenic forcing was applied following the CMIP5 design protocol from 1950 to 2005. RCP8.5 radiative forcing was used for future simulation from 2006 to 2100. Five simulations covering the historical period of 1850–1950 were performed to generate five different ocean states in 1950. Then, ten coupled ocean atmospheric simulations were run from each of these five historical simulations using randomly perturbed initial conditions (in 1950) to produce a total of fifty150-year simulations spanning the 1950–2100 period.

## Method

We mainly focus on the changes in the intensity and frequency of extreme precipitation under different global warming levels, namely 1.0°C, 1.5°C, 2.0°C, 3.0°C and 4.0°C above the preindustrial level. Since the large ensemble simulations span from 1950 to 2100, they are not able to calculate the GMST during the preindustrial period. However, a part of its contribution to CMIP5, CanESM2 produced historical simulations of a five-member ensemble for the 1850–2005 period. Therefore, temperature data from this set during 1860–1900 is used to estimate the GMST for the preindustrial era. The time series of GMST anomalies relative to the preindustrial level was smoothed with a nine-year moving average before selecting the first year when the GMST reached a specific warming level; this year and the two ten-year periods around it (a total of 21 years) were regarded as the future warming climate. The 21 years periods of the five warming climates were 1990–2010, 2004–2024, 2017–2037, 2038–2058 and 2056–2076. The 1.0°C warming level was used to represent the current climate since the current climate has warmed by approximately +1°C compared with the preindustrial period based on observations [20].

Many researchers have pointed out that GCMs exhibit biases in simulating the magnitude of extreme precipitation over China with overestimation over arid regions and underestimation over humid regions. Thus, it is necessary to correct any biases before using these models to conduct the projection research. If these biases are not removed, the future projection maybe unreliable. A bias-correction method based on quantile mapping was used to reduce such errors [21,22]. Quantile mapping techniques are among the most important and popular bias correction methods. First, we interpolated daily precipitation from CanESM2-LE to the 543 observational stations by using the inverse distance-weighted interpolation method. Then, we applied the local intensity scaling method to the daily precipitation data to ensure that the modeled wet-day frequency and intensity matched the wet-day frequency in observations during the calibration period [23]. Finally, the bias correction, based on quantile mapping, was independently applied to each station for each member. Here, we used the empirical quantile mapping, which empirically constructs maps between simulated and observed cumulative distribution functions. Due to the high climate sensitivity of CanESM2, the modeled warming is higher than observed warming. The 21-year period for which the modeled warming is closest to the observed warming during 1986–2005 was 1978–1998. Thus, we used modeled annual cycle during 1978–1998 as the calibration period for bias correction. The correction was applied (1) to the current climate (1.0°C warming) to evaluate the performance of bias correction in the observed 21 years period (1998–2018), and (2) to the future warming climate to produce bias-corrected daily precipitation data.

The generalized extreme value (GEV) distribution is widely used for modeling and characterizing extreme precipitation event [24–26]. The GEV distribution has been found to be suitable to fit the tails of the precipitation distribution conditions. Sufficient extreme event samples are required to give a more reasonable estimation of characteristics of the extremes by improving the fitting accuracy of extremes. The cumulative distribution function, F(z), is given by [27]:

$$F(z)=\text{exp}\{-{\left[\xi \left(\frac{\left(z-\mu \right)}{\sigma }\right)\right]}^{-\frac{1}{\xi }}\},\;1+\xi (\frac{z-\mu }{z-\mu })>0$$ (1)

where *μ*, *σ* and ξ are the location, scale, and shape parameters, respectively. These three parameters are estimated by the “L-moments” method, which is more effective and generates less uncertainty than other methods. In this study we used GEV to fit the annual maximum precipitation samples (total 21*50 = 1050) and calculated the return value of extreme precipitation with a long return period (e.g., 50 years and 100 years). The relative changes in the 50 years (100 years) return value can be used to demonstrate the intensity changes. The future return periods of the current 50-year and 100-year return values under different warming levels can be estimated to demonstrate changes in the frequency of extreme events. Future return periods shorter than 50 or 100 years imply a higher number of extreme events. We further examine the frequency changes of extreme precipitation by using the probability ratio (PR), which is defined as *PR* = *P*_1_/*P*_0_, where *P*_0_ represents the probability of a 50-year (*P*_0_ = 0.02) or 100-year (*P*_0_ = 0.01) return value during the current climate and *P*_1_ represents their probability under the future climate, expressed by the inverse of future return period. If *PR* > 1, the probability of extreme events in the current climate would increase under a future warming climate. The 95% confidence level of future PR over China in its six subregions can be calculated by a bootstrap scheme. Meanwhile, the impacts of extreme precipitation that were avoided at 1.5°C compared with a further warming climate were investigated using the formula below:

$$AI=\frac{C_{2}-C_{1.5}}{C_{2}}\times 100\%$$ (2)

where, *C*_1.5_ and *C*_2_ represent the changes in the 1.5°C warming climate and warmer climate, respectively.

## Results

### Performance of the CanESM2-LE

**Fig 2:**
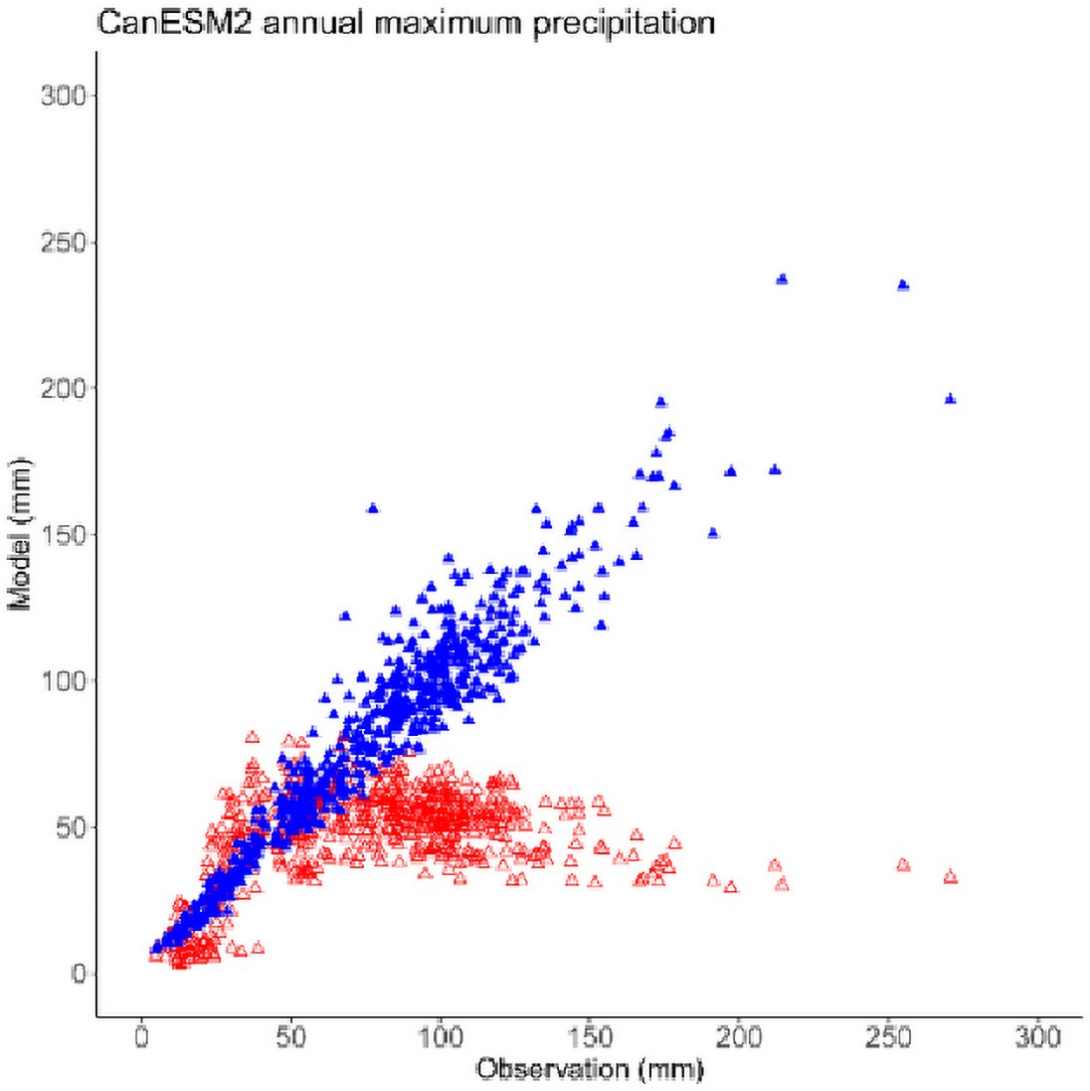
The correction between the observation data and CanESM2 modeling of annual maximum precipitation. The red triangle represents the raw ensemble and the blue triangle represents the bias-corrected ensemble.

**Fig 3:**
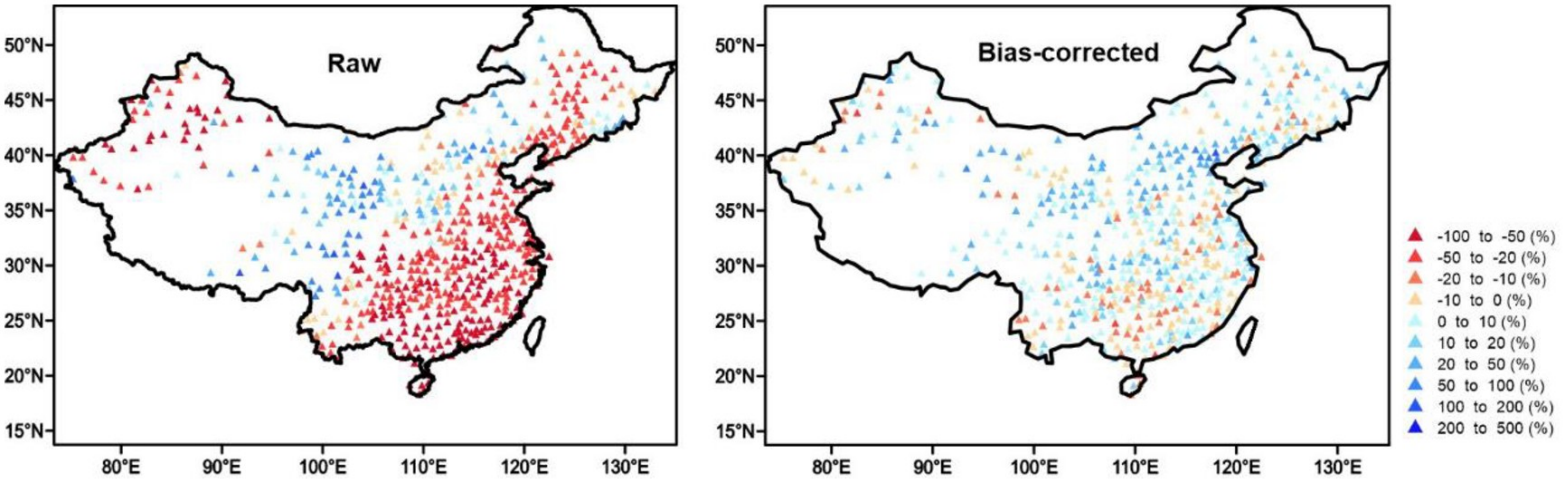
The spatial pattern of bias for climatology of annual maximum precipitation in the raw ensemble and the bias-corrected ensemble relative to observation during current climate conditions. (Made with Natural Earth. Free vector and raster map data @ naturalearthdata.com).

CanESM2 exhibits relatively large biases in simulating the annual maximum precipitation in terms of magnitude and spatial pattern. CanESM2-LE tends to underestimate the observed annual maximum precipitation with values larger than 50 mm (Fig 2). The regions with underestimation are mainly located over eastern China and northwestern China, while central China exhibits relatively weak positive biases. The negative biases over southeastern China can reach over 50% (Fig 3). The convective and microphysical parameterization schemes and coarse resolution of GCMs are responsible for these biases. Which have also been reported in CMIP5 models. The bias-corrected ensemble can sufficiently reproduce the characteristics of annual maximum precipitation with the biases reduced to no more than 20% across almost the entire region, which gives us confidence in the future projections.

### Changes in intensity of extreme precipitation events

**Fig 4:**
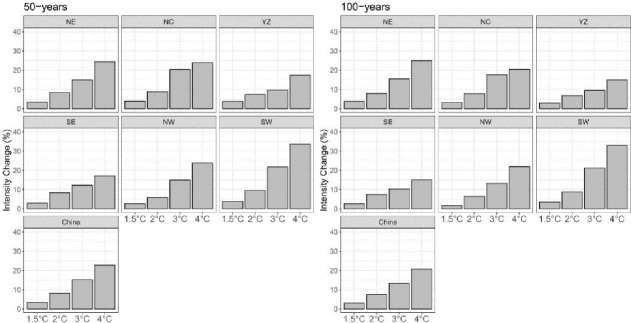
Regional average changes (relative to the current climate) in the 50-year and 100-year return values for China and its six subregions under 1.5°C, 2°C, 3°C and 4°C warming climates for CanESM2-LE.

Fig 4 shows the area-weighted average changes for 50-year and 100-year events under the four warming levels relative to the current climate across China and its six subregions. The intensity increases with global warming, with little difference between the two return periods under the same warming conditions. Taking all the regions together as an example, the 50-year (100-year) return value will increase by 3.5% (3.2%), 8.2% (8.0%), 15.2% (14.4%) and 22.8% (20.7%) under the four warming climates relative to the current climate. Southwestern China experiences the greatest increase in intensity in the future, with increase of nearly 4.0%, 9.6%, 21.7% and 33.7% under the four warming climates. Our study has demonstrated that regional average intensity of extreme precipitation increases at a rate of nearly 8% per 1°C warming, approximately the same rate as that of the moistening of the atmosphere due to global warming [28,29]. This is the main reason for the intensification of extreme precipitation. However, the intensity changes in some subregions over eastern China that are under the control of the East Asian summer monsoon (e.g., NC, YZ and SE) do not follow this scaling partly because the monsoon dynamics may be an important factor influencing the intensity of extreme precipitation.

### Changes in the frequency of extreme events

**Fig 5:**
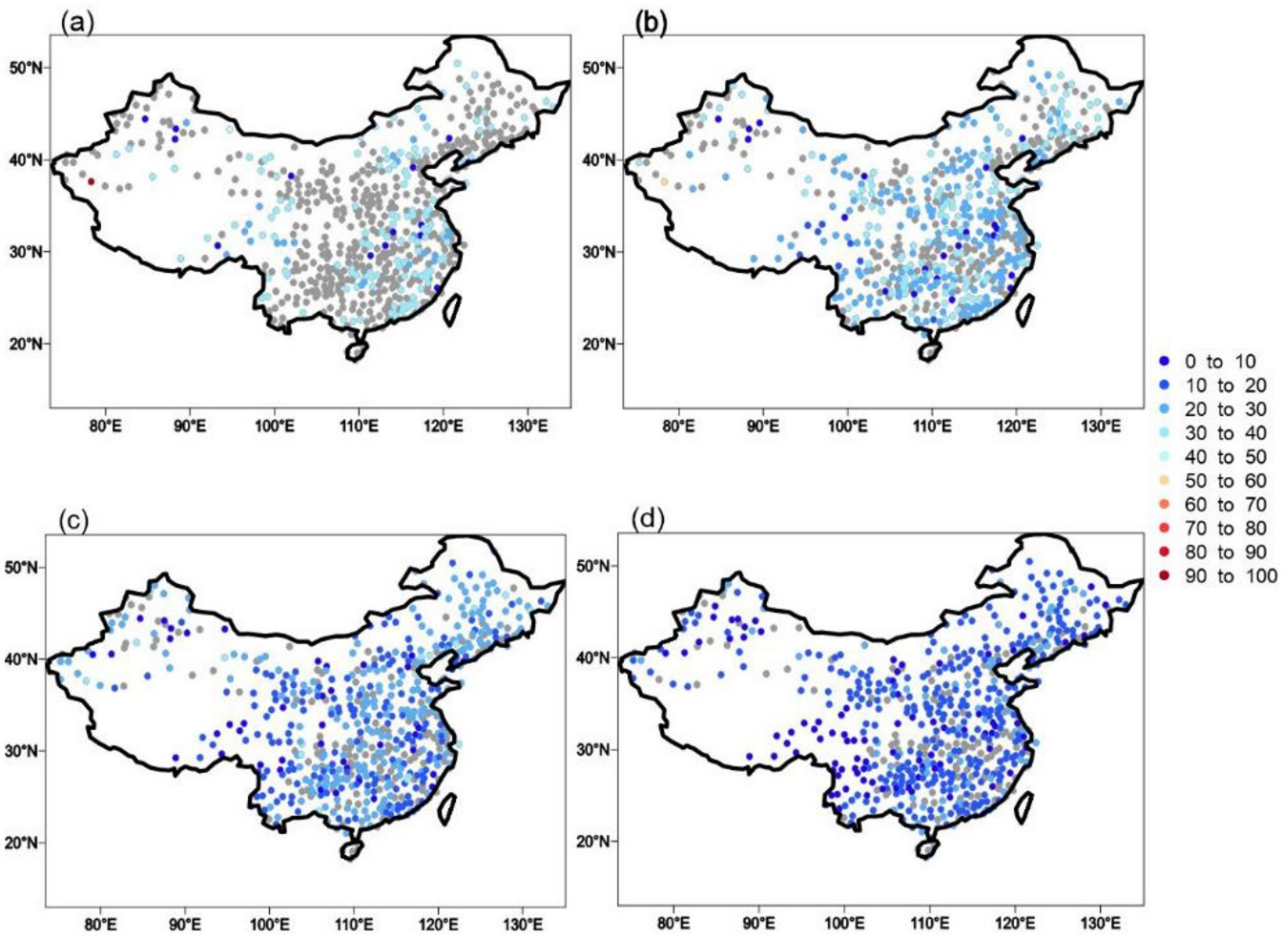
The projected return period of current extreme events with a 50-year return period under 1.5°C (a), 2°C (b), 3°C (c) and 4°C (d) warming climates. The changes in return period that are not significantly at the 5% significant level are represented by gray dots. (Made with Natural Earth. Free vector and raster map data @ naturalearthdata.com).

**Fig 6:**
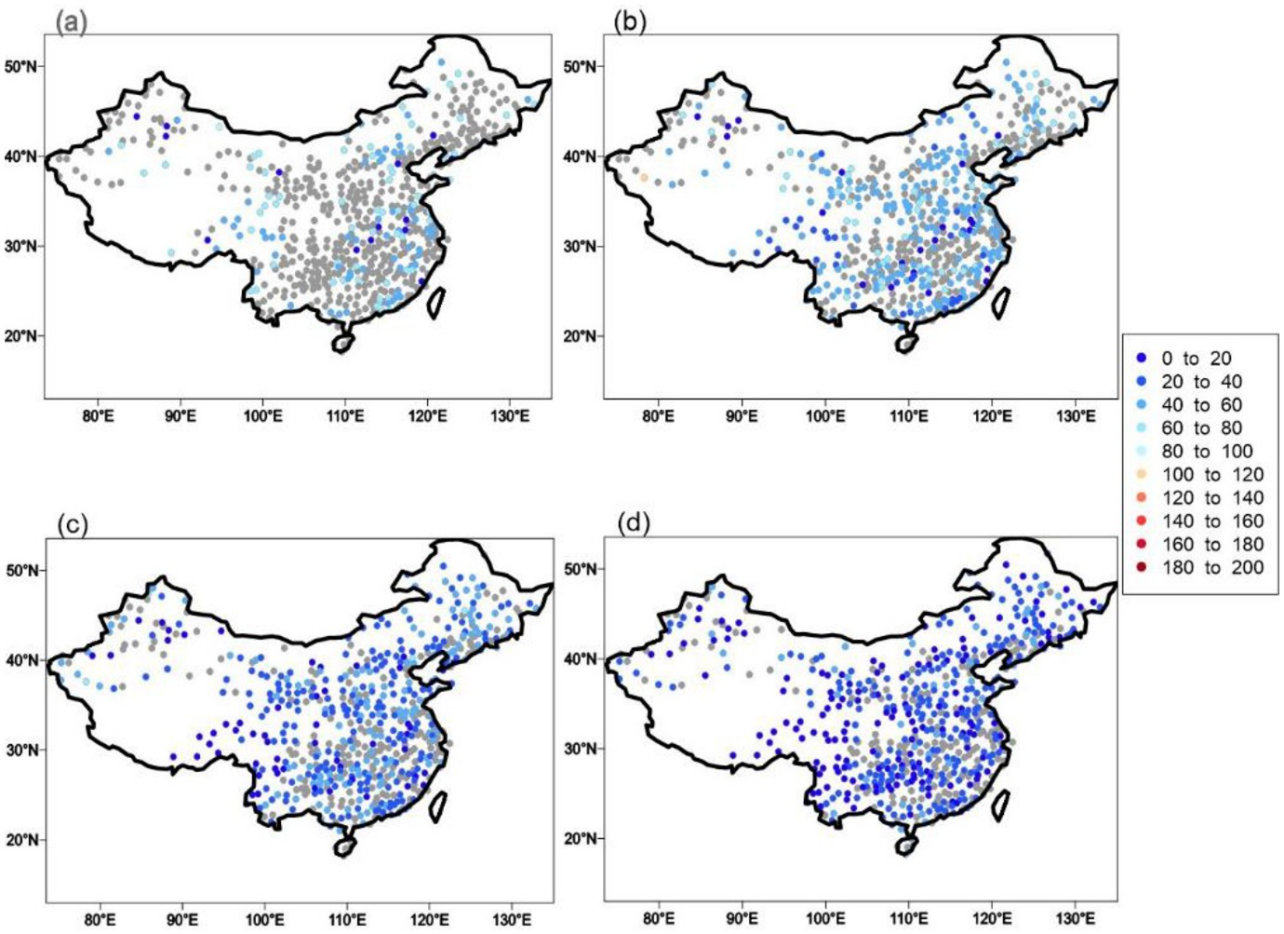
Same as Fig 5, but for 100-year extreme precipitation events. (Made with Natural Earth. Free vector and raster map data @ naturalearthdata.com).

Fig 5 shows the pattern of the future return period for the current 50-year events under different warming levels. Blue (red) dots represent return periods of less (more) than 50 years, implying that the frequency of current extreme events will increase (decrease). We can see that the frequency of extreme precipitation events at most stations clearly increases under a 1.5°C warming climate with a return period ranging from 40 to 50 years. However, nearly one third of stations show significant change in the frequency of extreme events. The frequency of 50-year extreme events continues to increases when GMST continues to warm, especially under high warming levels. The proportion of stations with significant change also increase with warming. For example, the future return period at most stations ranges between 40 and 50 years under a 1.5°C warming climate. While the values at most stations are less than 30 years under a 4°C warming climate, those at some stations over southwestern China are less than 10 years. This suggests that the extreme events expected once every 50 years under the current climate are expected to occur approximately every 10 years under a climate warming by 4°C over southwestern China. The distribution of the precipitation events within a 100-year return periods is similar to that of the 50-year return period, with only some differences in magnitude (Fig 6). We also calculate the median return period across the whole region to show the changes in the frequency of extreme precipitation events on a national level. The events that are expected to occur every 50 years (100 years) in the current climate are expected to occur approximately every 41 years (82 years), 32 years (62 years), 22 years (42 years) and 15 years (29 years), respectively, under four warming levels.

**Fig 7:**
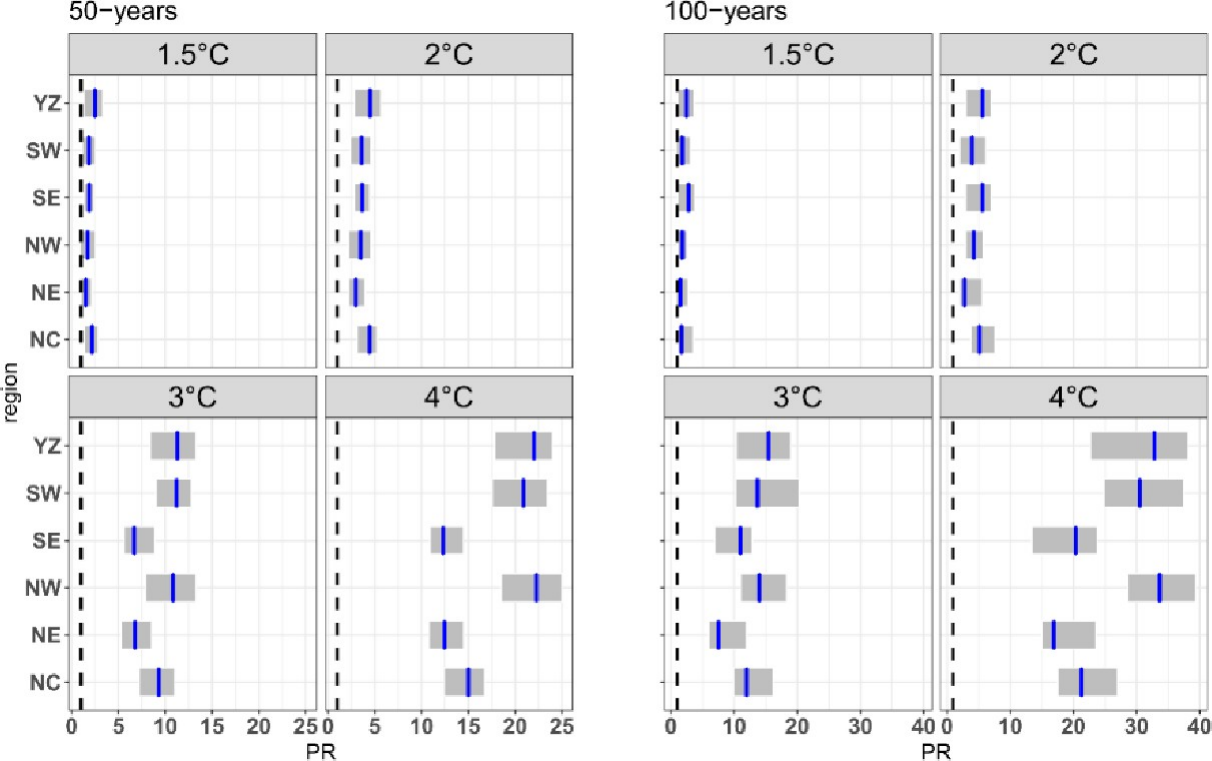
PR of 50-year and 100-year extreme precipitation events occuring under four warming levels across China and its six subregions.

The risk ratios of the current 50-year and 100-year extreme precipitation events over the six subregions under the four warming levels are analyzed in Fig 7. All six regions will experience more extreme precipitation events in the future, especially under high warming levels. The PR of 50-years events of extreme precipitation in the current climate over the six subregions exhibit little difference between 1.5°C and 2.0°C. However, NW, SW and YZ exhibit the greatest increase in PR under the future climate scenario. The risk of extreme precipitation events expected to occur once every 50 years will be almost 11 (21) times more likely to occur under a 3.0°C (4.0°C) warming climate. The uncertainty of future PR is also larger under high warming conditions than under low warming conditions. The future PR, with 95% confidence levels, of current 50-year extreme events occurring over YZ are (1.3, 3.4), (2.8, 5.7), (8.4, 13.3) and (17.8, 24).

Compared with 50-year events, the risk associated with 100-year events will increase in the future. That is, the rarest extreme events (longest return period) exhibit the largest increase in risk, especially under high warming levels [30,31]. Taking as an example the three subregions with the largest increases in PR (YZ, SW and NW), the risk of extreme precipitation events expected to occur once every 100 years will be almost 14 (32) times more likely to occur under a 3.0°C (4.0°C) warming climate. This implies that the events expected every 100 years in the current climate will occur every 7 (3) years if the GMST warms by 3.0°C (4.0°C). Additionally, the uncertainties associated with longer return periods will be larger. The 95% confidence level of extreme events over YZ is (17.8, 24) for 50-year events and (22.7, 38) for 100-year events.

**Fig 8:**
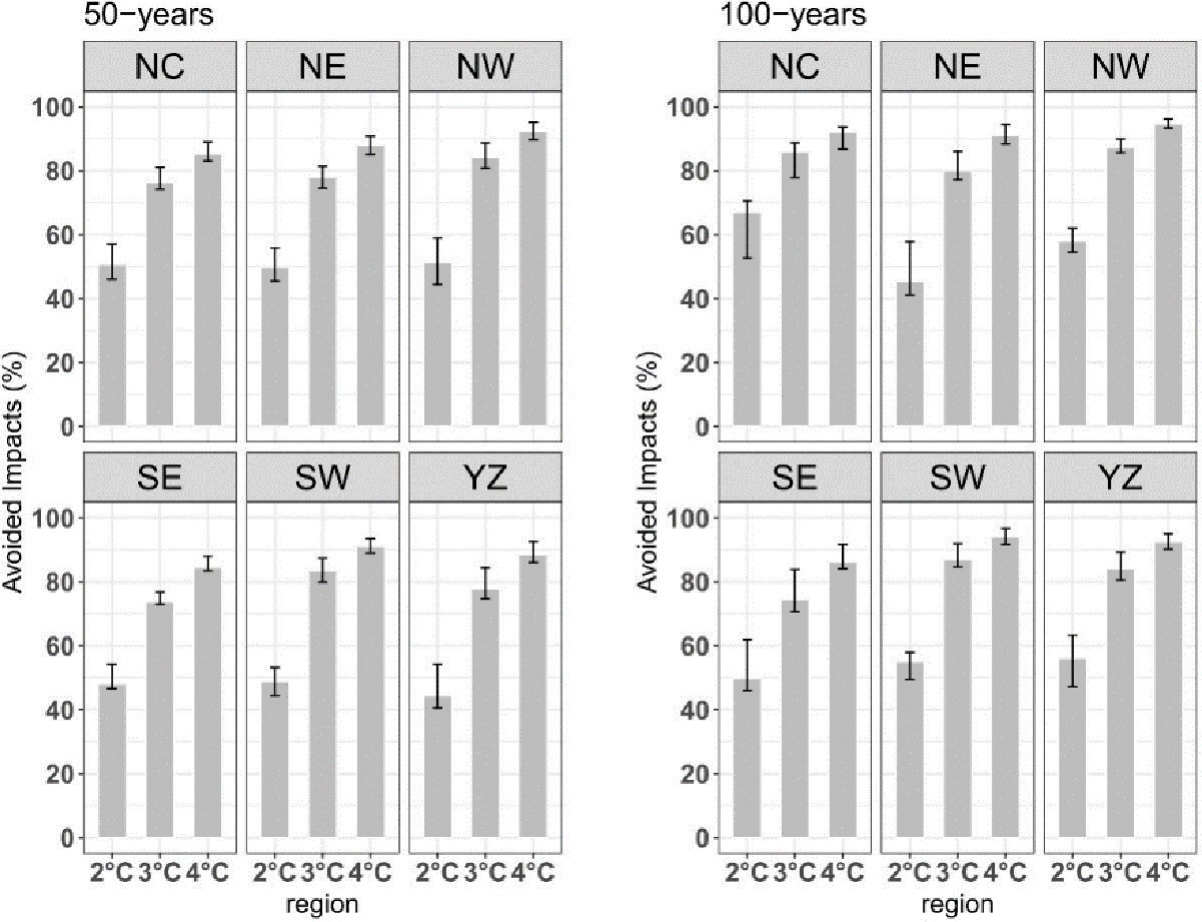
The avoided risk of extreme precipitation events with 50-year and 100-year return period over the six subregions of China at 1.5°C warming compared with warmer future scenario (units: %). The black vertical lines over the bars show the 95% confidence interval.

Furthermore, we used formula (2) to quantify the impacts avoided in the 1.5°C warming climate compared with further warmed climates (by 2.0°C, 3.0°C and 4.0°C) climates (Fig 8). Limiting warming below 1.5°C will help avoid approximately 40–50%, 70%80%, and over 90% of the increase in the risk of extreme events in almost all subregions that would be expected if the GMST continues warming to 2.0°C, 3.0°C and 4.0°C, respectively. The uncertainties of avoided impact decrease with global warming.

## Conclusion

This paper used a large ensemble simulation from CanESM2 (CanESM2-LE) to project the change in intensity and frequency of extreme precipitation events (events with 50-year and 100-year return periods) under future warming, providing a new insight from single GCM’s large ensemble simulation. A bias-correction method based on quantile mapping was first applied to daily precipitation data during the current climate for each member of the ensemble to evaluate its performance. Then future changes in extreme precipitation under different warming levels were projected over China and its six subregions. The results show the following:

1. The raw ensemble exhibits a poor performance in simulating annual maximum precipitation over China, with negative biases over southern and western China and positive biases over central China. The bias-corrected ensemble sufficiently reproduces the observed magnitude and pattern of annual maximum precipitation in the current climate, with biases between -20% and 20%.
2. The intensity of extreme precipitation events will increase as the GMST rises. For China as a whole, the regional average intensity of extreme precipitation increases at a rate of nearly 8% per 1°C warming, approximately the same rate as that of the moistening of the atmosphere due to global warming, although there may be differences between different subregions.
3. The frequency of extreme precipitation events also increases with global warming. The rarer events show a higher increase in risk, especially under high warming climate conditions. Three subregions, YZ, SW and NW, received the greatest increase in risk of extreme precipitation events under the future warming climate. The current 50-year events are almost 22 times more likely to occur under the 4°C warming climate, implying that the extreme precipitation events expected occur once every 50 years under the current climate will occur approximately every 2 years under 4°C warming.

Our results are based on a large ensemble, which may also be model dependent. The future projection includes three sources of uncertainties: 1) model uncertainty; 2) uncertainty in scenario; 3) uncertainty in internal variability. The dominant source of uncertainty depends on the variable and region under consideration. For China, as an example, many researchers have indicated that model uncertainties are an important concern in the future projection of extreme precipitation [21,32]. Thus, more large ensemble simulations from other models are needed to understand the uncertainties stemming from the model structure. In addition, the ensemble used in the present research produces only future simulations under the RCP8.5 scenario, and it is therefore not possible to study the uncertainty that stems from different emission scenarios. Large ensembles resulting from dynamical downscaling with finer resolution and improved deep convection parameterization are expected to give more accurate projections of extreme precipitation at the regional scale [33,34].
